# Editorial: Sustainable Solutions in Food Technology

**DOI:** 10.3389/fnut.2022.855521

**Published:** 2022-02-23

**Authors:** Rui M. S. Cruz, Golfo Moatsou, Jooyeoun Jung

**Affiliations:** ^1^Department of Food Engineering, Institute of Engineering, Universidade do Algarve, Faro, Portugal; ^2^MED - Mediterranean Institute for Agriculture, Environment and Development, Faculty of Sciences and Technology, Universidade do Algarve, Faro, Portugal; ^3^Department of Food Science and Human Nutrition, Agricultural University of Athens, Athens, Greece; ^4^Department of Food Science and Technology, Oregon State University, Corvallis, OR, United States

**Keywords:** food, sustainability, waste recovery, green technologies, packaging

The sustainability of the food system is one of the key goals for a sustainable society. The world population is growing, as well as the carbon footprint of the technologies associated with the processing of food products. On one hand, these technologies try to guarantee the population's demands with more food, but on the other hand, use a huge amount of resources such as water and energy, contributing to a negative environmental impact. However, there is a new trend regarding consumers' concern about climate change and healthier lifestyles. Consumers are changing their diet and food options, demanding natural and healthier food products, associated with cleaner and more sustainable technologies. To address these challenges, novel strategies must be studied and applied to develop food with high nutritional quality and safety. This must be done whilst using less resources, thus contributing to an ecologic economy. This Research Topic consists of seven articles that provide recent advances and insights in new technologies and food sources that guarantee food quality and safety whilst also having a positive environmental impact.

The article of Mbye et al. is a comparative study of the effects of pasteurization (65 °C for 30 min and 75 °C for 30 s) and high-pressure processing (HPP 350, 450, and 550 MPa for 5 min at 4 °C) of camel and bovine milk on the physicochemical features, proteolysis, texture profile, and microstructure of fresh cheese. The effects of the treatments on the cheese textural profile were different between the camel and bovine cheeses; while heat treatment at 65 °C for 30 min resulted in the hardest bovine milk cheese, HPP treatment at 350 MPa for 5 min gave the highest value for camel milk cheese. The hardness of the cheeses was associated with low yield and moisture content.

Ibrahim et al. showed the application of interactive and intelligent packaging for fresh fish shelf-life monitoring. A high qualified duplex laminated package with a nano-encapsulated pH monitoring label for fresh fish was printed. The interactive Quick Response code icon was combined in a designed package with important information about cooking, smart packaging, and fish quality. Particle size, zeta potential, and surface area are measured for a nano-encapsulated indicator which exhibits 74.4 nm, 23.6 mV, and 88.9 m^2^/g, respectively: overall migration, water vapor, and oxygen permeability. The properties of printing for 11 color spots were evaluated by x-rite before and after the cold storage period without any detectable changes in the rate of color change. There was a good agreement between microbial count and smart indicator color change. The microbial growth in packaged fish was suppressed up to 48 h and the bacterial and fungal counts were acceptable for up to 6 days-storage at 4 °C. Moreover, the migration from the duplex laminated PE/PET packaging ranged from 0.3 up to 1.3 mg/dm^2^ with limits of acceptance up to 10 mg/dm^2^. The oxygen gas permeability and the water vapor transmission rate were low at a humidity of 50% and water vapor saturation of 80%, respectively. The authors suggest that the packaging system developed in this study can also contribute to the reduction of product waste and to the improvement of consumer information and awareness.

Adaro et al. presented the biosynthesis of a novel antibacterial dipeptide, using proteases from south American native fruits, useful as a food preservative. Soluble granulosain showed lower catalytic potential in all liquid-liquid biphasic media than in the same buffer. However, 50% (v/v) ethyl ethanoate in 100 mM Tris(hydroxymethyl)aminomethane hydrolchloride buffer pH 8.0 allowed the expression of the highest catalytic potential of both soluble enzymes. In 50% v/v ethyl ethanoate, soluble antiacanthain and granulosain catalyzed the synthesis of Z-Tyr-Val-OH with 72 ± 0.15 and 60 ± 0.10% maximal peptide yields, respectively. Multi-point immobilization in glyoxyl-silica did not lead to better peptide yields than soluble enzymes, in that liquid-liquid biphasic medium under the same reaction conditions. Soluble and glyoxyl-silica immobilized antiacanthain in almost anhydrous ethyl ethanoate (Xw: 1 × 10^−5^) were able to retain 17.3 and 45% of the initial proteolytic activity of antiacanthain in 100 mM Tris hydrolchloride buffer pH 8.0, respectively, at 40 °C under agitation. Soluble and glyoxyl-silica immobilized granulosain were inactivated under the same reaction conditions. Glyoxyl-silica immobilized antiacanthain showed to be a robust biocatalyst in almost anhydrous ethyl ethanoate (Xw: 1 × 10^−5^), eliciting the best peptide yield (75 ± 0.13%). The synthesis reaction of Z-Tyr-Val-OH could not proceed when soluble antiacanthain was used under the same conditions. Both peptidases only catalyzed the synthesis reaction under kinetic control, using activated acyl donor substrates. This work reports a novel broad-spectrum antibacterial peptide that significantly decreased the specific growth rates of Gram-positive and Gram-negative microorganisms at very low concentrations; contributing to a new safe food preservative applicable for different food systems.

Dorado et al. showed the analysis and potential value of compounds extracted from Star Ruby, Rio Red, and Ruby Red grapefruit, and grapefruit juice processing residues *via* steam explosion. In this study, pectic hydrocolloids, sugars, volatiles, phenolics, and flavonoids were extracted from Star Ruby, Rio Red, and Ruby Red GP, and WG using a continuous pilot-scale steam explosion system. Up to 97% of grapefruit juice oils and peel oils could be volatilized and contained 87–94% d-limonene. The recovery of pectin, as determined by galacturonic acid content, was between 2.06 and 2.72 g/100 g. Of the phenolics and flavonoids analyzed in this study, narirutin and naringin were extracted in amounts of up to 10,000 and 67,000 μg/g, respectively.

The study of Ibáñez et al. showed the antimicrobial effect of a proteolytic enzyme from the fruits *of Solanum granuloso-leprosum* (Dunal) against *Helicobacter pylori*. All the strains tested were susceptible to granulosain I with MIC from 156.25 to 312.5 μg/mL and MBC from 312.5 to 625 μg/mL, respectively. Besides, all the strains tested were susceptible to the RAP with MIC from 312.5 to 625 μg/mL and MBC from 625 to 1,250 μg/mL, respectively. The effect of granulosain I and RAP on the transcription of *H. pylori* genes encoding pathogenic factors, omp18, ureA, and flaA, with respect to a housekeeping gene (16S rRNA), was evaluated by RT-PCR technique. Granulosain I and RAP significantly decreased the expression of pathogenic factors: omp18, ureA, and flaA. The combined inhibitory effect of granulosain I or RAP and an antibiotic such as amoxicillin (AML, 10 μg), clarithromycin (CLA, 15 μg), levofloxacin (LEV, 5 μg), and metronidazole (MTZ, 5 μg) was evaluated, using the agar diffusion technique. Granulosain I and RAP showed a significant synergistic effect on AML, CLA, and LEV, but no significant effect on MTZ was observed. Besides, granulosain I and RAP did not show toxicological effects at the concentrations studied. Finally, granulosain I and RAP could be used as safe natural food additives and as adjuvants for conventional therapies against *H. pylori*.

Marques et al. presented omega-3 fatty acid fortification of flax through nutri-priming. To address these goals, brown flax was nutri-primed in three priming solutions, control [0% fish oil (FO)], 10% FO, and a 20% FO solution, and determined the FA content and profile of seeds and sprouts and germination percentage of primed seeds. n-3 FA nutri-priming with FO altered the FA profile in seeds and sprouts, with increases in the absolute content of 20:5 n-3, 22:6 n-3, 22:5 n3, 18:4 n-3, and 20:4 n-6. However, n-3 FA nutri-priming did not increase the absolute content of 18:2 n-6, 18:3 n-3, total saturated FA, total monounsaturated FA, total polyunsaturated FA, total n-6 FA, or total n-3 FA. The results also showed that n-3 nutri-priming decreased the germination percentage of primed seeds, with 10 and 20% FO priming solution reducing germination by 4.3 and 6.2%, respectively. Collectively, n-3 nutri-priming modified the n-3 FA profile in flax; however, the process does not increase the total n-3 FA content and inhibits germination of primed seeds. Further research utilizing different seed types, oil types, and oil concentrations needs to be conducted to fully determine if n-3 nutri-priming is a commercially viable approach for n-3 fortification of seeds and sprouts.

Jahan et al. assessed the effect of the partial replacement of dietary fish meal with fermented or untreated soybean meal (FSM and SM) on the growth, hematology, antioxidant activities, gut morphology, and digestive enzyme activities of juvenile silver barb, *Barbonymus gonionotus*. Five different diets were fed to the fish two times daily for 90 days. After 90 days of feeding trial, FM 40, FM 20 + FSM 20, and FM 20 + SM 20 diet groups showed significantly higher weight gain (WG) and specific growth rate (SGR) compared to the FSM 40 and SM 40 diets. Hepatosomatic index (HSI) and viscerosomatic index (VSI) were significantly higher in fish fed with the FSM 40 and SM 40 diets than those of fish fed with the FM 40 diet. Hematocrit, hemoglobin, and erythrocyte count were significantly lower in fish fed with the SM 40 diet compared to fish fed with the FM 40 and FM 20 + FSM 20 diets. Superoxide dismutase and catalase activities in the liver were significantly higher in fish fed with the SM 40 diet compared to fish fed with the FM 40 diet. However, serum thiobarbituric acid reactive substances in fish fed with the experimental diets were unaltered. Fish showed a significant reduction of villus height (Vh) in the anterior and posterior intestine of fish fed with the FSM 40 and SM 40 diets, whereas muscular thickness was opposite to the findings of Vh. Digestive enzyme activities in the intestine were significantly higher in fish fed with the FM 40 diet compared to those in the SM 40 diet. The results of the present study revealed that the 50% of FM can be replaced by FSM or SM as a source of protein. The authors suggest that their study can be an experimental model to assess the effect of SM in the diet of other omnivores including monogastric farm animals.

## Author Contributions

RC, GM, and JJ contributed to manuscript writing, revision, and read. All authors contributed to the article and approved the submitted version.

## Conflict of Interest

The authors declare that the research was conducted in the absence of any commercial or financial relationships that could be construed as a potential conflict of interest.

## Publisher's Note

All claims expressed in this article are solely those of the authors and do not necessarily represent those of their affiliated organizations, or those of the publisher, the editors and the reviewers. Any product that may be evaluated in this article, or claim that may be made by its manufacturer, is not guaranteed or endorsed by the publisher.

